# Designing Semiconductor Nanowires for Efficient Photon Upconversion via Heterostructure Engineering

**DOI:** 10.1021/acsnano.2c04287

**Published:** 2022-07-25

**Authors:** Mattias Jansson, Fumitaro Ishikawa, Weimin M. Chen, Irina A. Buyanova

**Affiliations:** † Department of Physics, Chemistry and Biology, Linköping University, SE-58183 Linköping, Sweden; ‡ Graduate School of Science and Engineering, Ehime University, 790-8577 Matsuyama, Japan

**Keywords:** nanowires, upconversion, solar cells, photonics, heterostructures

## Abstract

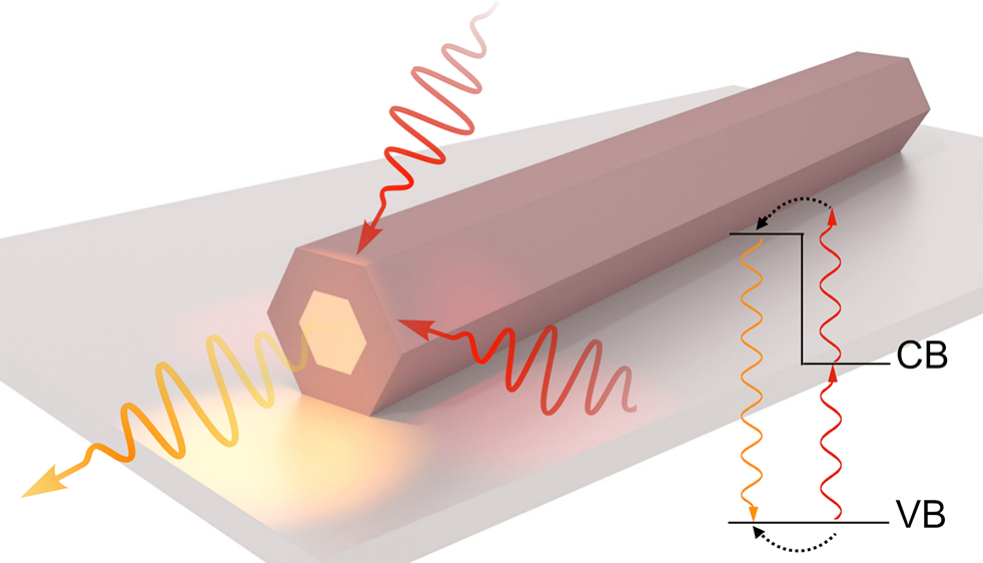

Energy upconversion via optical processes in semiconductor nanowires (NWs) is attractive for a variety of applications in nano-optoelectronics and nanophotonics. One of the main challenges is to achieve a high upconversion efficiency and, thus, a wide dynamic range of device performance, allowing efficient upconversion even under low excitation power. Here, we demonstrate that the efficiency of energy upconversion via two-photon absorption (TPA) can be drastically enhanced in core/shell NW heterostructures designed to provide a real intermediate TPA step via the band states of the narrow-bandgap region with a long carrier lifetime, fulfilling all the necessary requirements for high-efficiency two-step TPA. We show that, in radial GaAs(P)/GaNAs(P) core/shell NW heterostructures, the upconversion efficiency increases by 500 times as compared with that of the constituent materials, even under an excitation power as low as 100 mW/cm^2^ that is comparable to the 1 sun illumination. The upconversion efficiency can be further improved by 8 times through engineering the electric-field distribution of the excitation light inside the NWs so that light absorption is maximized within the desired region of the heterostructure. This work demonstrates the effectiveness of our approach in providing efficient photon upconversion by exploring core/shell NW heterostructures, yielding an upconversion efficiency being among the highest reported in semiconductor nanostructures. Furthermore, our work provides design guidelines for enhancing efficiency of energy upconversion in NW heterostructures.

Photon energy upconversion, that is, a process in which several low-energy photons are converted into a high-energy photon, is of significant importance in a wide variety of research fields. In biological imaging and labeling, it provides superior sensitivity in combination with deep penetration, low phototoxicity, and the ability to perform imaging in vivo.^1−4^ In medicine, it facilitates drug delivery allowing remotely controlled drug release.^5,6^ Photon upconversion also provides a means for infrared light detection and visualization^7,8^ and can be used in integrated photonic applications, such as microscale wavelength-division multiplexing,^9^ and in unconventional pumping schemes in optoelectronic devices to achieve, for example, upconversion lasing.^9−11^ Another application area, which is of particular interest in the quest for efficient renewable energy generation, is the possibility of using upconversion materials for solar energy harvesting, for example, in solar-driven water splitting schemes and third-generation photovoltaic devices.^12−17^ Here, upconversion allows harvesting low-energy photons that cannot be absorbed through one-photon absorption via band-to-band transitions in a light absorber, thereby potentially improving device efficiency above the Shockley–Queisser limit.

Upconversion at the nanoscale has long been studied in, for example, lanthanide-doped nanocrystals and in triplet–triplet annihilator (TTA) molecules,^18^ which have demonstrated high upconversion efficiencies. However, these material systems have some inherent drawbacks. For example, since the photon absorption occurs between discrete atomic or molecular states, their spectral absorption bandwidth and energy tunability are limited.^13^ Semiconductor nanostructures, such as nanowires (NWs) from III–V compounds and related alloys, represent an important class of upconverting materials that are particularly desirable for applications in nano-optoelectronics and photonics. These structures offer strong light absorption within a small material volume^19^ as well as a wide energy tunability thanks to a sizable library of available compounds and alloys further assisted by quantum confinement effects at the nanoscale. They also allow electrical detection. Moreover, eased constraints in lattice matching within NWs and between NWs and their substrate materials open the avenue for integration of optically efficient III–V materials with Si^20−22^ as well as fabrication of high-quality NW heterostructures from highly mismatched materials, thereby extending the functionality of the structures. The III–V NWs can also have superior nonlinear optical properties^23−25^ desirable for designing highly efficient upconverters. Upconversion via multiple photon absorption in such structures has mainly been demonstrated via second-harmonic generation,^8,23−29^ though two-photon absorption (TPA) was also reported.^27^ Since, in these processes, the upconversion occurs through a virtual intermediate state, they generally require very high excitation densities, which restricts the range of practical applications. This requirement may be relaxed, however, when TPA takes place via a real intermediate state—a process often referred to in the literature as a two-step two-photon absorption (TS-TPA) process. In III–V NWs, the TS-TPA via defect states,^30,31^ or via quantum dots, embedded in NWs^32^ has been reported. However, to date, the reported upconversion efficiency in III–V nanostructures^30−33^ as well as other semiconductor nanostructures^34^ remains relatively low, typically <0.1%, especially at low excitation powers.^13^

In this work, we attempt to push the limit of low-power upconversion efficiency in semiconductor nanostructures, through the approach of radial heterostructure engineering of NWs by exploring radial core/shell NWs with a nitrogen free III–V core and a dilute-nitride shell of a lower bandgap with a favorable band alignment between the core and shell. Dilute nitrides, obtained from parental III–V materials by substitution of a few percent of group-V atoms with nitrogen (N), have a number of attractive properties promising for optoelectronic, photovoltaic, and spintronic applications.^29,35−42^ A large difference in size and electronegativity between the N atom and the replaced group-V host atom dramatically affects the electronic structure of the forming alloy: It leads to a giant decrease in the bandgap energy, which can be as much as 270 meV/%N,^43^ caused by a dramatic down-shift of the conduction band (CB) edge upon N incorporation, while the valence band (VB) edge remains practically unaffected.^44^ Using dilute-nitride alloys in such nanostructured NWs is expected to facilitate easy and wide-range tuning of the band alignment at the heterointerface thanks to this N-induced giant down-shift of the CB states, which broadens and extends the usable range of the primary light wavelength. It could also prolong the carrier lifetime at the real intermediate state, taking advantage of the strong carrier localization effect well-known to this class of highly mismatched alloys.^40^ In this work, we show that these properties indeed allow efficient energy upconversion to the wide-bandgap core states when an intermediate step of the TPA process involves the band states of the narrow-bandgap shell. Design rules for optimization of this process are also established, based on in-depth experimental studies of TS-TPA in GaAs/GaNAs and GaAsP/GaNAsP NW heterostructures combined with a rate equation analysis.

## Results and Discussion

**Figure 1:**
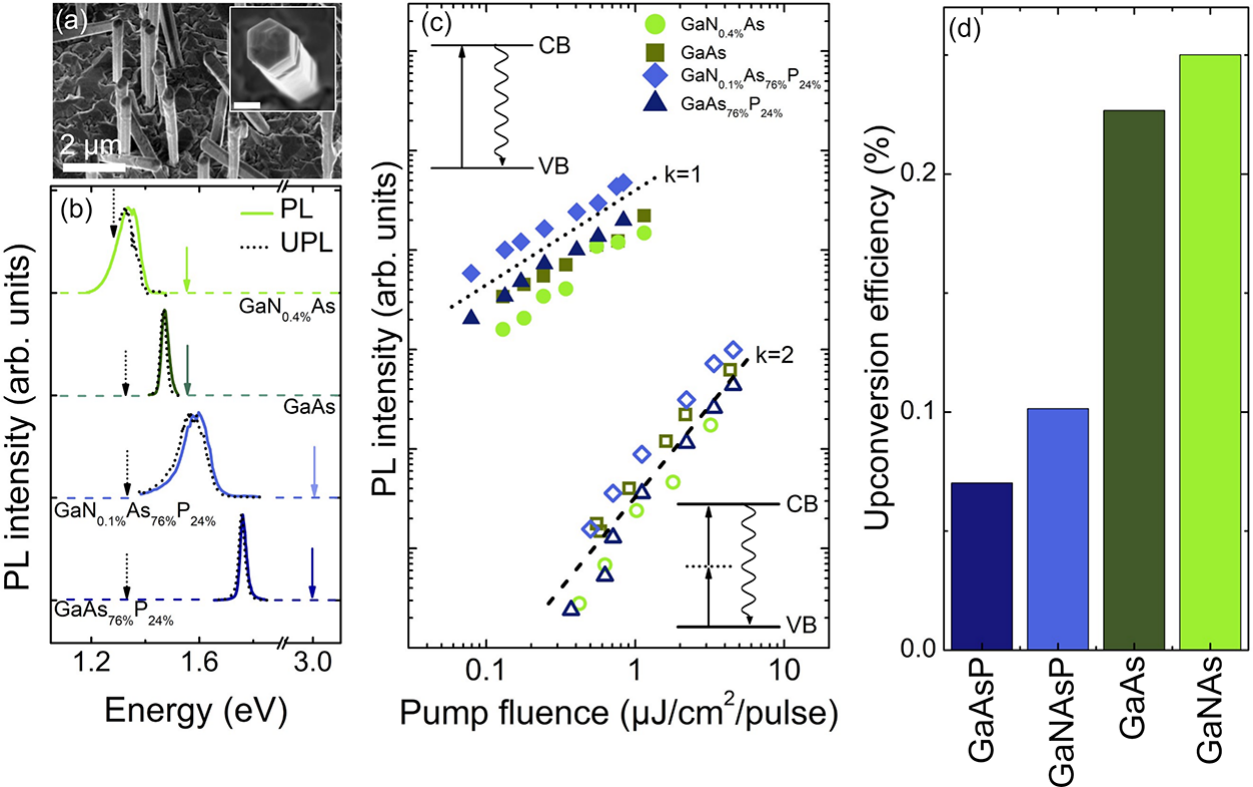
TPA upconversion through a virtual state. (a) An SEM image of the GaNAs NW array. The inset shows a top-view image of a single NW, where the scale bar is 200 nm. (b) PL (the solid lines) and UPL (the dotted lines) spectra acquired at 7 K from various NWs under pulsed excitation. The solid and dashed arrows indicate the excitation energies during the PL and UPL measurements, respectively. (The band gap energies of the NWs are given in Table 3.) All spectra are normalized to the same maximum intensity and are offset vertically for clarity. The UPL spectrum acquired from the GaNAs sample is cut off at the low-energy side due to the proximity to the excitation laser. (c) Integrated PL (the solid symbols) and UPL (the open symbols) intensity as a function of the excitation pump fluence (*P*). The dotted (dashed) line outlines a linear (quadratic) power dependence of the emission intensity. The upper inset illustrates the scheme of excitation (the straight arrow) and the PL (the waved arrow) process, whereas the lower inset corresponds to that involving the UPL process. (d) The TPA upconversion efficiency of the studied materials measured at *P* = 0.8 μJ/cm^2^/pulse.

The investigated N-free and N-containing III–V NWs were grown by molecular beam epitaxy (MBE) on (111) Si substrates. They include uniform GaAs, GaAsP, and GaNAsP NWs grown in a vapor–liquid–solid (VLS) mode and radial core/shell GaAs/GaNAs and GaAsP/GaNAsP NW heterostructures. The latter contain a dilute-nitride shell (GaNAs or GaNAsP) fabricated via vapor–solid growth and a VLS-grown core of a parental N-free material (GaAs or GaAsP). In order to understand the effects of material composition on the upconversion processes, structures with different N, As, and P content were investigated. Details of the growth conditions and structural parameters of the investigated NWs can be found in the Experimental Section. All NWs were found to form dense arrays containing 2–4.5 μm long wires with diameters ranging from ∼100 nm for the uniform structures to ∼400 nm in the core/shell heterostructures; see Figure 1a where a scanning electron microscopy (SEM) image of the GaAs/GaNAs NW array is shown as an example. Representative SEM images of the other NW samples can be found in Section S1 of the Supporting Information.

### Two-Photon Absorption

Before discussing TS-TPA in the NW heterostructures, we first analyze linear and nonlinear optical properties of the constituent materials. Figure 1b provides an overview of the photoluminescence (PL) spectra of different dilute-nitride alloys and their parental materials studied in this work. (The structural parameters and band gap energies of these structures are given in Table 3.) Under the above bandgap excitation through one-photon absorption, that is, a linear process, the PL spectra (the solid lines) are dominated by a near-band-edge emission caused by recombination of excitons trapped within the band tail states. The PL spectra experience a red shift upon N incorporation, which is caused by a N-induced decrease in the bandgap energy due to the well-known giant bandgap bowing in dilute nitrides.^35−46^ Simultaneously, spectral broadening of the PL emission upon N incorporation is observed, which is typical for dilute nitrides and is determined by an energy distribution of the localized states.^40,45,46^ The same emissions, though significantly weaker, can also be excited when the excitation photon energy (*hυ*_exc_) is tuned below the bandgap (*E*_g_). The corresponding spectra of such upconverted PL (UPL) emission are shown in Figure 1b by the dotted lines, where the excitation energies are marked by the dotted arrows. Similarity of the emission spectra under both above- and below-bandgap excitation conditions suggests that the same radiative transitions are involved. This in turn proves that free carriers in all studied materials can be generated by below bandgap (or anti-Stokes) photons due to energy upconversion. (A small red shift of the UPL spectra is attributed to a much lower carrier density generated under anti-Stokes excitation, leading to a reduced state filling.)

In order to understand the upconversion mechanism, we investigated dependences of the integrated PL intensity (*I*) on the pump fluence (*P*) under the one-photon and anti-Stokes excitation. The corresponding results are shown in Figure 1c by the filled and open symbols, respectively. Both dependences can be approximated by a power function *I* ∝ *P*^*k*^, though the power index *k* changes from *k* = 1 for the one-photon excitation with *hυ*_exc_ > *E*_g_ to *k* = 2 for the anti-Stokes excitation with *hυ*_exc_ < *E*_g_. The linear power dependence is typical for excitonic transitions under conventional above-bandgap excitation. On the other hand, the observed change to the quadratic power dependence of the PL intensity under the anti-Stokes conditions implies that the generation of the photoexcited carriers now occurs via a nonlinear optical process, such as TPA, a third-order nonlinear process involving virtual states.^47^ The relevant optical transitions are schematically illustrated in the upper and lower insets of Figure 1c, respectively.

We now compare the efficiency of the TPA process between the studied materials. Figure 1d compiles their upconversion efficiency (UCE), which was calculated as the ratio between the integrated UPL and PL intensities (here, the displayed UCE values are measured under an identical excitation fluence of *P* = 0.8 μJ/cm^2^/pulse). First of all, it is seen that the UCE values clearly increase with increasing As content, which could be attributed to a higher TPA coefficient of GaAs as compared with GaP.^48^ More surprisingly, the TPA process seems to be promoted in dilute nitrides, implying an increase in the third-order susceptibility upon nitrogen incorporation. Though the exact physical mechanism behind this effect requires further studies, we note that N-induced enhancement of the second-order susceptibility tensor and, thus, the second-harmonic generation efficiency has previously been reported in GaNP NWs^29^ and was attributed to effects of symmetry breaking due to local disorder and mixing of CB states in dilute nitrides. A similar mechanism could be relevant to our case. We should note that alloy compositions are the decisive factors in determining the TPA efficiency of the studied NWs, whereas the orientation and thickness of the NWs plays a minor role as explained in Section S2 of the Supporting Information. The observed enhancement of the nonlinear response in the dilute-nitride NWs is beneficial for potential applications in nonlinear nanophotonics.

### TS-TPA in Core/Shell Heterostructured NWs

**Figure 2:**
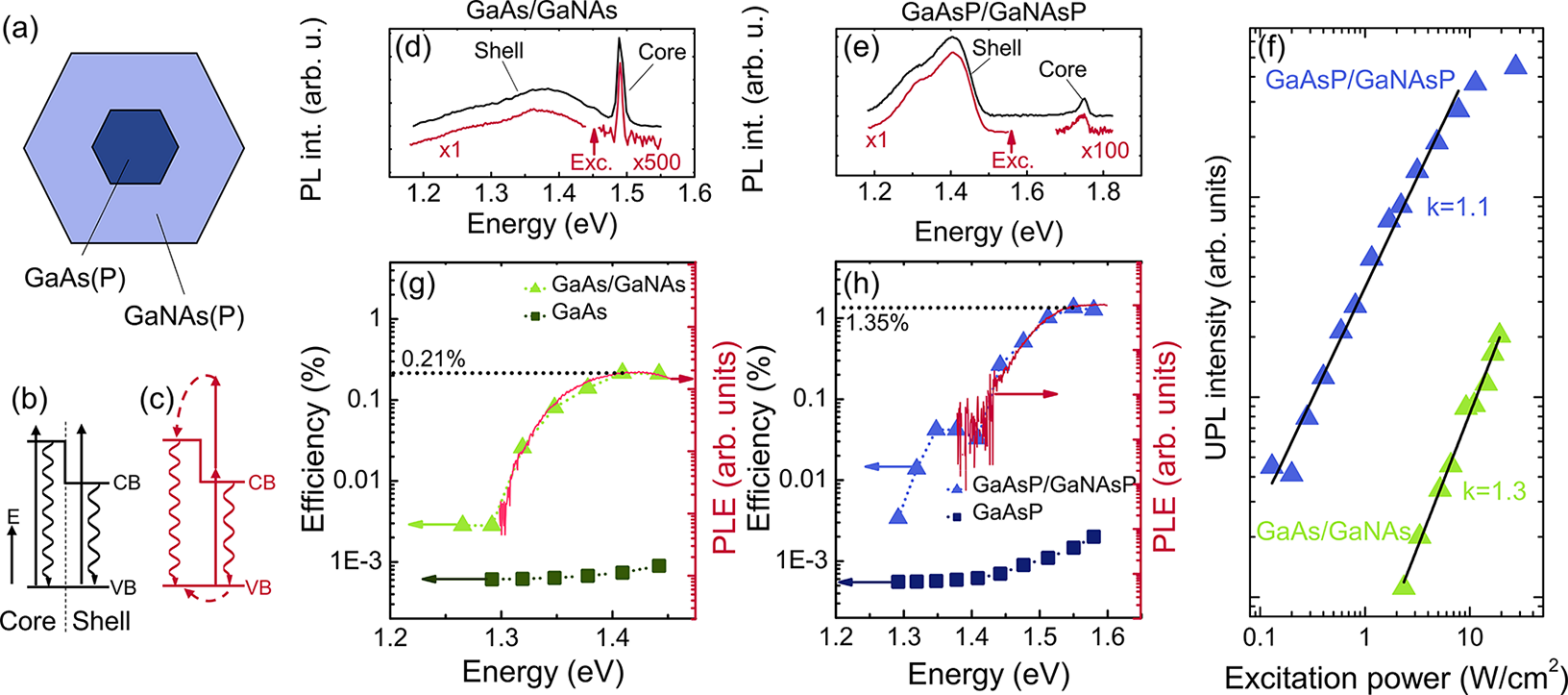
TS-TPA upconversion in core/shell NW heterostructures. (a) A schematic view of the cross section of the studied NW heterostructures. (The GaNAs-based NWs have an additional outer GaAs capping layer which is not shown in the figure.) (b, c) Electronic structure of the core/shell heterostructure as well as carrier generation (the straight arrows), recombination (the wavy arrows), and transfer (the curved dashed arrows) processes under one-photon (b) and TS-TPA (c) excitation. (d, e) Emission spectra measured from the GaAs/GaNAs (d) and GaAsP/GaNAsP (e) core/shell NWs when the excitation photon energy is tuned above (the black curves) and below (the red curves) the core bandgap (the band gap energies of the structures are given in Table 3). The UPL spectra of the core are scaled as indicated in the figure. The arrows labeled as “Exc” indicate the excitation energy in the UPL measurements. The PL measurements under the one-photon excitation were performed with the excitation energy of 1.75 eV (d) and 2.33 eV (e). (f) Integrated UPL intensity (the symbols) as a function of excitation power. The lines represent the best fit to the data using the power function with the power factor *k* as indicated in the figure. (g, h) Spectral dependences of the upconversion efficiency in the core/shell heterostructures (the triangles) and the reference sample of N-free uniform NWs (the squares). The red curves show PLE spectra of the shell emission under the one-photon excitation. All measurements were performed at 7 K.

Due to the quadratic power dependence of the TPA via virtual states presented above, the efficiency of this process is strongly power dependent and becomes reasonably high only under pulsed light excitation with high pumping powers (see Figure 1c). The efficiency of this process in the studied NWs decreases by several orders of magnitude under continuous-wave (cw) excitation with a power density identical to the time-integrated excitation power during the pulsed excitation conditions, which clearly is a drawback for applications such as photovoltaics. It is known^31^ that more efficient energy upconversion under low-power cw excitation is achievable via TS-TPA, that is, when an intermediate state involved in the photon absorption is a real state. Such conditions should be possible to fulfill in dilute-nitride-based heterostructures, for example, in a core/shell NW heterostructure with a dilute-nitride shell and a nitrogen-free core (Figure 2a). The band alignment in such a heterostructure is shown schematically in Figure 2b,c, reflecting the fact that the bandgap reduction in dilute nitrides chiefly occurs due to a downshift of the CB edge.^44^ We note that radial band bending induced by an electric field due to, for example, a piezoelectric effect and surface states, is not shown in Figure 2b,c for simplicity. We believe that its effect on photon upconversion efficiency is negligible in our NWs in view of our experimental observation that the upconversion efficiency is nearly independent of excitation power, since the degree of screening of an electric field is expected to vary under different excitation power levels (see also Section S6 of the Supporting Information).

We now consider possible light absorption and emission processes in such a structure, which are represented in Figure 2b,c by the straight and wavy arrows, respectively. If the excitation photon energy exceeds the bandgap of the wide-bandgap NW core (Figure 2b), the photogeneration of charge carriers could occur in both the core and shell regions, giving rise to two peaks in the PL spectra. The TS-TPA process depicted in Figure 2c becomes possible when *hυ*_exc_ is tuned between the bandgaps of the core (*E*_g_^core^) and shell (*E*_g_^shell^) regions. It involves the following steps: (i) electron and hole generation in the shell region; (ii) absorption of a second photon by the photogenerated electron in the shell accompanied by its transfer to the larger-bandgap core; and (iii) diffusion of the photogenerated hole from the shell to the core without additional photon absorption. (Note that the sequencing between the processes (ii) and (iii) could depend on excitation power and could be reversed.) The radiative recombination of the electrons and holes transferred from the shell to the core constitutes the UPL.

PL measurements performed under cw excitation on two types of the core/shell NW heterostructures, GaAs/GaNAs with [N] = 0.3% (Figure 2d) and GaAsP/GaNAsP with [P] = 24% and [N] = 1.1% (Figure 2e), confirm the aforementioned scenario. Under the conditions of *hυ*_exc_ > *E*_g_^core^ (the band gap energies of these structures are given in Table 3), the PL spectra (the black curves) contain two peaks, corresponding to the radiative recombination transitions in the core and shell layers. The core emission can also be detected under anti-Stokes excitation (the red curves). Moreover, the UPL intensity (shown by symbols in Figure 2f) now exhibits a much weaker dependence on the excitation power, which can be approximated by a power function with *k* = 1.3 and 1.1 for the GaAs/GaNAs and GaAsP/GaNAsP NWs, respectively. This suggests that the monitored upconversion process occurs via a real state, that is, a TS-TPA process. For such a process, *k* may take any value between 1 and 2 depending on the lifetime of the intermediate state, as compared with *k* = 2 expected for upconversion through a virtual state.^31^ From Figure 2f, we also note that the UPL can be observed down to very low excitation powers (*W*_exc_), as low as 0.1 W/cm^2^.

The origin of the intermediate state acting as a stepping stone in the TS-TPA can be identified by measuring the UCE as a function of the excitation energy. The corresponding results are shown by the triangles in Figure 2g,h for the GaNAs-based and GaNAsP-based heterostructured NWs, respectively. It is found that the UCE in both structures exhibits a strong dependence on *hυ*_exc_ that closely resembles PL excitation (PLE) spectra of the shell emission (the red curves), which reflect the generation of free carriers in the dilute-nitride shell due to one-photon absorption. Therefore, the huge UCE enhancement by more than 2 orders of magnitude when *hυ*_exc_ > *E*_g_^shell^ provides clear evidence that the intermediate states in the TS-TPA process are the CB states of the shell. As expected, absence of such states in the reference samples of uniform GaAs and GaAsP NWs leads to a much lower UCE for all excitation energies (the squares), where TPA through a virtual state is the dominant process.

In the TS-TPA process observed in our dilute-nitride-based core/shell NWs when *E*_g_^shell^ < *hυ*_exc_ < *E*_g_^core^, the key lies on the two-step excitation of electrons due to the specific band alignment of the heterostructures, as shown in Figure 2c. This is because, due to the flat VB alignment across the heterojunction, a photoexcited hole is expected to easily diffuse from the small bandgap shell to the larger bandgap core without requiring the involvement of a second photon. For a CB electron in the shell generated by the first photon, on the other hand, a second photon is required to further excite it above the CB edge of the core (being a hot electron) to overcome the energy barrier for electron transfer from the shell to the core. The efficiency of this transfer and, thus, the TS-TPA process are governed by the competition between momentum/energy relaxation of the hot electrons back down to the CB edge of the shell and transfer of the hot electrons to the core.^49,50^ Considering that the charge transfer occurs at the core–shell heterointerface, it should be rather efficient in NW heterostructures with a large interface-to-volume ratio. Several effects may further assist this charge transfer in the studied NW heterostructures. First, the diffusion of the photoexcited holes may lead to a radial electric field, causing a drift of the hot electrons from the shell to the core. Second, the charge transfer at the heterointerface is facilitated if the electrons in the shell CB participating in absorption of the second photon are trapped by localized states so that their wave function contains nonzero values of the wavevector.^51,52^ This condition is satisfied in the dilute-nitride shell due to the N-induced electron localization known to exist in such alloys. Furthermore, due to a finite penetration of the electron wave function in the shell into the core, there is a certain probability that the electron transfer from the shell to the core could be regarded as spatially quasi-direct, resulting in a larger transfer coefficient.

**Figure 3:**
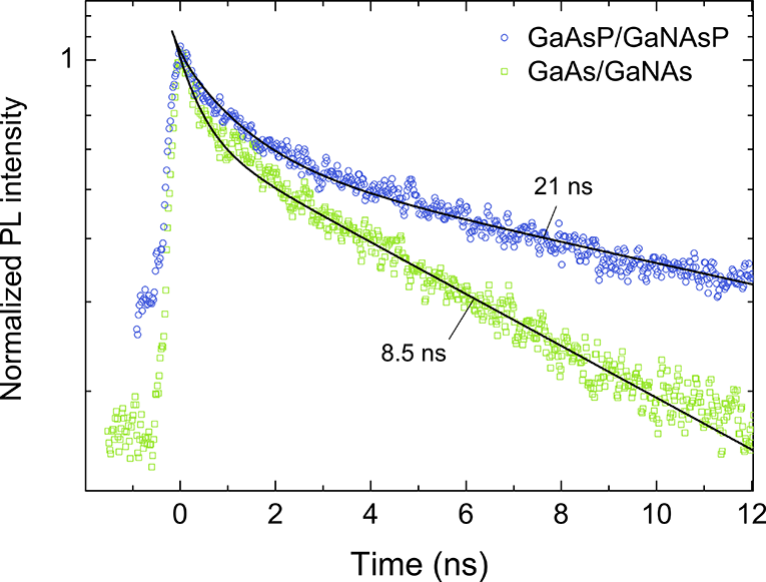
Transient PL response. Temporal profiles of the integrated shell emission measured at 7K from the GaAs/GaNAs (the green squares) and GaAsP/GaNAsP (the blue circles) core/shell NWs, under the excitation wavelength of 410 nm. The solid lines show the best fit to the data by a biexponential function, with the fast/slow lifetimes of 0.4/8.5 ns for the GaAs/GaNAs NWs and 1.3/21.0 ns for the GaAsP/GaNAsP NWs.

By comparing results of Figure 2g,h, we note a significant difference in the TS-TPA efficiency between the GaAs/GaNAs and GaAsP/GaNAsP NWs, with the maximum values of 0.21% and 1.35%, respectively. To understand its origin, we further examine optical properties of the shell materials using transient PL measurements. In both structures, the PL decay of the shell emission (the symbols in Figure 3) can be fitted (the solid lines) by a biexponential function which contains slow and fast decay components (see Section S3 of the Supporting Information for a detailed analysis). Such behavior is typical for dilute-nitride NWs and likely reflects contributions of radiative transitions from the regions with distinctly different lifetimes, determined by a combined effect of radiative and nonradiative recombination. The latter process can dominate in the NW regions with a high density of defects acting as efficient nonradiative recombination centers, for example, surface states of the NW, interfacial defects at the core/shell NW heterojunction, and point or structural defects.^53−55^ The overall PL decay, however, is significantly slower in the case of GaNAsP, which is primarily caused by two factors. First of all, it reflects a longer lifetime of the slow decay component in GaNAsP (21 ns) as compared with the GaNAs material (8.5 ns). This could be attributed to the known decrease of the oscillator strength of the optical transitions and, therefore, an increase of the radiative lifetime when phosphorus is introduced into the alloy.^56^ In addition, the fast PL decay component is less pronounced in the GaNAsP NWs, which may stem from (i) a lower density of such defect regions with large nonradiative recombination rates and (ii) a shorter diffusion length in the material, reducing an impact of the defect regions.

### A Rate Equation Analysis of TS-TPA

**Figure 4:**
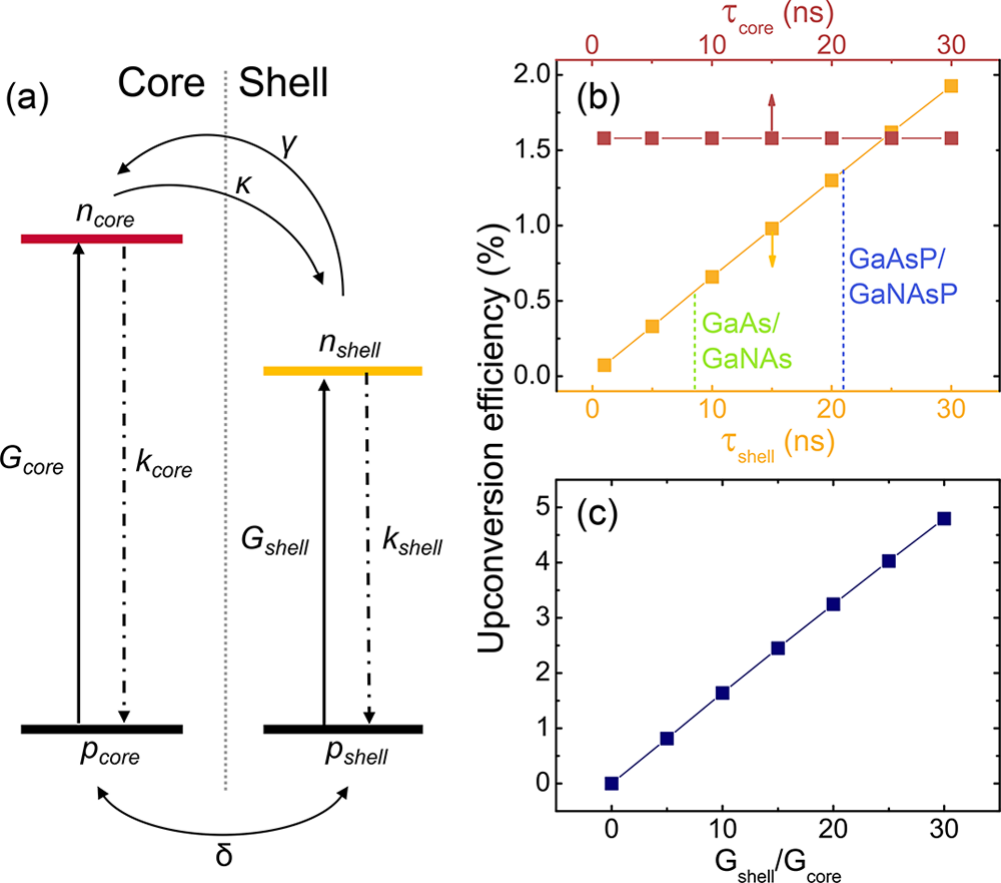
Rate-equation model for carrier generation, transfer, and recombination. (a) A schematic diagram of the model. *n*_shell/core_ and *p*_shell/core_ denote the electron and hole concentrations in the shell/core regions, whereas *G*_shell/core_ and *k*_shell/core_ represent generation and recombination in these regions. The terms γ, κ, and δ describe the upconversion and back transfer of electrons and the transfer of holes across the heterojunction, respectively. (b) The simulated UCE as a function of the lifetimes of the shell (the yellow symbols) and the core (the red symbols). The dashed vertical lines indicate measured lifetimes of the dominant slow PL decay component in the specified NW heterostructures. (c) The simulated UCE as a function of the ratio of the generation rates between the shell and core regions.

To investigate whether the observed increase in the shell PL lifetime with phosphorus incorporation can explain the observed higher UCE in the corresponding NW heterostructures, we model the excitation-transfer-recombination processes using a rate equation model depicted schematically in Figure 4a. It is assumed that the electron (*n*) and hole (*p*) concentrations in the core and shell of the NW are governed by the generation (*G*) and recombination coefficients (*k*) and by the terms γ, κ, and δ, which correspond to the rate of upconversion and back-transfer of the electrons and transfer of holes across the heterojunction, respectively. It is also assumed that free carrier generation occurs solely due to light absorption, which is reasonable considering that the investigated structures were undoped and the measurements were performed at 7 K. A suitable set of rate equations with minimum complexity is then given as
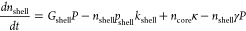
1
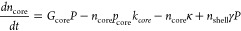
2
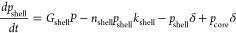
3
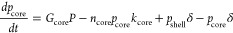
4where *P* is the simulated excitation power.

The parameters *k*_core_ and *k*_shell_ describe the transient response of the core and shell emissions and can, therefore, be deduced from the measured PL lifetimes. Moreover, the *G*_shell_*/G*_core_ ratio affects the intensity ratio of the core and shell emissions and can, thus, be determined based on the PL spectra of Figure 2d,e. Since no energy barrier is expected to exist for the holes at the core/shell interface, the parameter δ is chosen to be large, simulating the case that the holes can freely cross the interface. Finally, from simulations we see that κ does not affect the UCE and, therefore, was set to zero. The γ term is chosen to yield the best match between the simulated and experimentally measured UCE values of Figure 2g,h. The simulation parameters, which give the best agreement between the simulation and experiments, are given in Table 1.

**Table 1:** Simulation Parameters Yielding the Best Agreement between the Simulation and Measurements for the Two Studied NW Heterostructures

structure	*G*_shell_/*G*_core_	*k*_core_ (cm^–3^ s^–1^)	*k*_shell_ (cm^–3^ s^–1^)	γ (s^–1^)	κ (s^–1^)	δ (s^–1^)
GaAsP/GaNAsP	8.1	10^–3^	4 × 10^–4^	0.6 × 10^–3^	0	10
GaAs/GaNAs	1.8	1.5 × 10^–2^	4 × 10^–3^	1.1 × 10^–3^	0	10

We can now establish impacts of different parameters on the upconversion efficiency. Figure 4b shows the simulated UCE as a function of the core (red) and shell (yellow) PL lifetimes, τ_core_ and τ_shell_, using the other parameters given in Table 1 for the GaAsP/GaNAsP NWs. It is found that the core lifetime does not affect the UCE, since a change of τ_core_ equally affects both the PL and UPL intensities. In contrast, a strong increase of the UCE is observed with increasing shell lifetime (see also Section S4 of the Supporting Information). For example, the UCE increases from 0.63% to 1.35% when the τ_shell_ is changed from 8.5 to 21 ns, that is, between the experimentally determined PL lifetimes of the GaNAs and GaNAsP shell (indicated in Figure 4b by the dashed lines). It is clear that the longer PL lifetime in the GaNAsP shell can be an important factor that boosts the UCE in the related heterostructures. We, therefore, conclude that the lifetime of the intermediate state in the TS-TPA process is of vital importance for the upconversion efficiency. Considering this result, we emphasize the benefit of using dilute-nitride materials in the NW heterostructures as these alloys have an inherently longer radiative lifetime than the parental N-free direct bandgap materials due to N-induced mixing of the CB states.^57^ For example, the radiative lifetime of GaNAs was found to be more than three times longer than that of GaAs.^45^

The rate equation simulations also shows that the ratio *r* = *G*_shell_*/G*_core_ greatly affects the upconversion efficiency (Figure 4c). Here, *r* can be increased by promoting carrier generation in the shell and, therefore, provides an additional degree of freedom in optimizing upconversion by engineering an electric field distribution of the excitation laser light inside the NWs.

### Effects of the Electric Field Distribution

**Figure 5:**
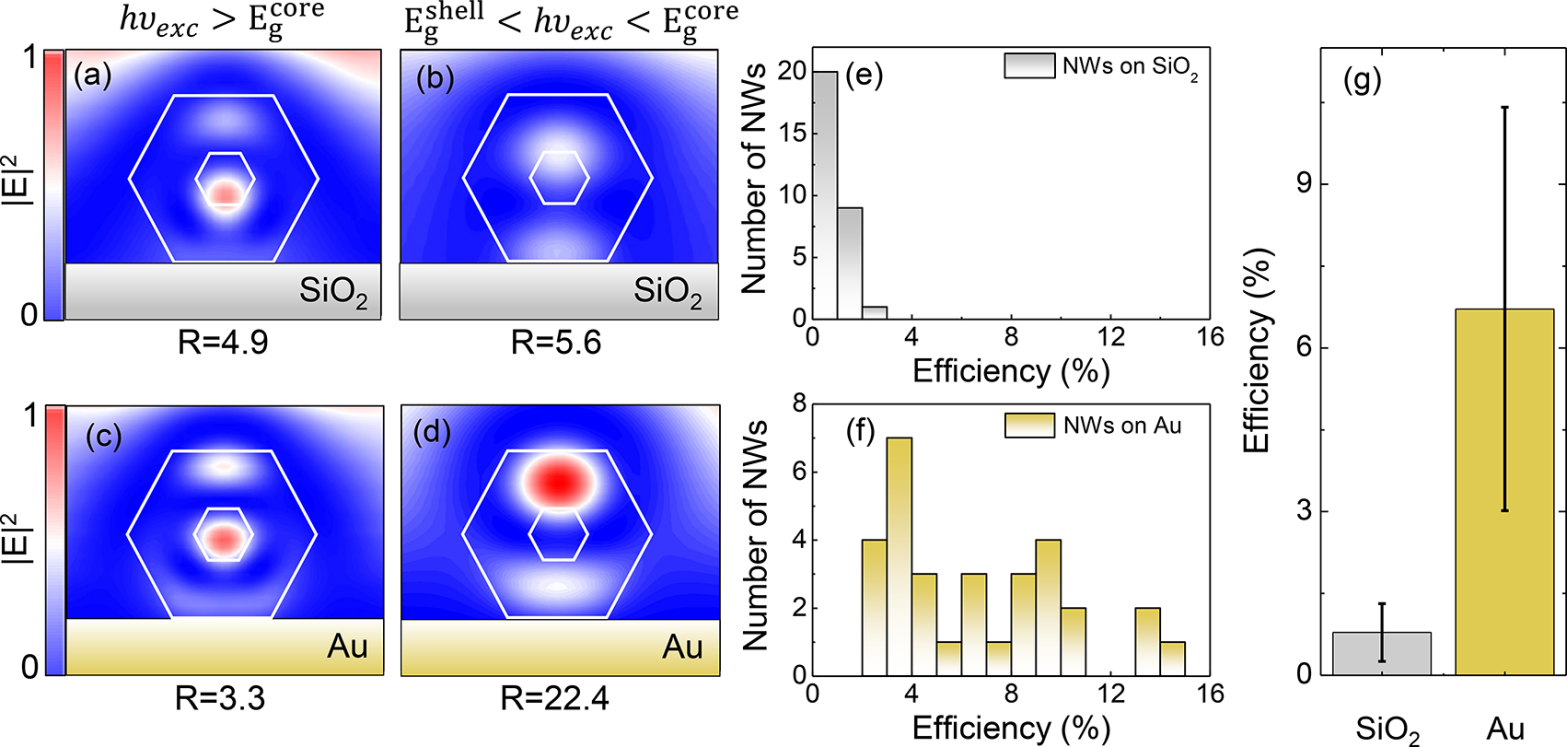
Effects of the electric field distribution. (a–d) Simulated |*E*|^2^ intensity for GaAsP/GaNAsP NWs on SiO_2_ (a, b) and gold (Au) (c, d) substrates. The simulation results are shown for two photon energies of 2.33 and 1.55 eV, which correspond to the one-photon excitation with *hυ*_exc_ > *E*_g_^core^ (a, c) and the TS-TPA excitation with *E*_g_^shell^ < *hυ*_exc_ < *E*_g_^core^ (b, d). The white lines outline the core and shell regions of the NW. (e, f) Histograms of the UCE measured from 30 individual GaAsP/GaNAsP NWs on SiO_2_ (e) and Au (f) substrates, with *hυ*_exc_ of 2.33 and 1.55 eV for the PL and UPL measurements, respectively. (g) The mean value of UCE in the NWs on the two substrates, where the error bars indicate a standard deviation from the mean value. The measurements were performed at 5 K.

It is well-known that the dielectric environment, for example, a substrate material, can have a large impact on the electric field distribution of the excitation light inside a NW^58−60^ and, therefore, on the carrier generation. To understand and optimize the corresponding effects, we perform finite-difference time-domain (FDTD) simulations of the electric field distribution of excitation light in a GaAsP/GaNAsP NW lying on a SiO_2_ (Figure 5a,b) and a gold (Figure 5c,d) substrate. Since nitrogen is not expected to significantly modify the refractive index,^60^ GaAsP material parameters were used for the entire NW. The field distribution is computed for two photon energies of 2.33 and 1.55 eV, which correspond to the one-photon excitation with *hυ*_exc_ > *E*_g_^core^ (Figure 5a,c) and TS-TPA excitation with *E*_g_^shell^ < *hυ*_exc_ < *E*_g_^core^ (Figure 5b,d), respectively. Light confinement within the NW shell is quantified by calculating the ratio , where numerical integration of the squared electric field |*E*|^2^ is performed over the shell and core cross-section areas (*A*). The simulations show that in the NW placed on a SiO_2_ substrate the electric field distribution does not change significantly between these excitation conditions: *R* = 4.9 and 5.6 for *hυ*_exc_ = 2.33 and 1.55 eV, respectively. In contrast, in the case of a gold substrate, *R* increases from 3.3 to 22.4 when *hυ*_exc_ is tuned from above to below the core bandgap. This shows that by placing a NW on a gold substrate, the excitation photons responsible for the TS-TPA process can be better confined within the shell region, which should promote the UCE of this process. In the FDTD simulations, another advantage of the hybrid NW-on-gold structures also becomes apparent, namely a generally higher concentration of the excitation laser light in the NW. This effect occurs under both excitation conditions and, therefore, should enhance all processes leading to carrier generation in the structures (see Section S5 of the Supporting Information for details).

To verify this experimentally, we placed 30 GaAsP/GaNAsP NWs on each substrate, measured the PL and UPL intensity, and computed the upconversion efficiency of the individual NWs (Figure 5e,f). First of all, it was found that the PL intensity is on average 3 times greater when NWs are placed on a gold substrate as compared to those on a SiO_2_ substrate. An even greater increase by 24 times is observed for the UPL intensity. Under the one-photon excitation, this enhancement reflects a stronger concentration of the laser light in the NW-on-gold structures combined with reflection of the emitted light from gold, which leads to a higher collection efficiency. Under the TS-TPA excitation, however, improved confinement of the laser light within the shell region (i.e., the higher *R* ratio) should also be responsible for the increased UPL intensity, by boosting the upconversion efficiency. Indeed, we found that while most of the NWs on the SiO_2_ substrate have the UCE below 2%, it exceeds this value in all NW-on-gold structures, reaching 15% in some of the NWs. On average the upconversion efficiency in the NW-on-gold structures is found to be 8 times higher than that on the SiO_2_ substrate (Figure 5g), confirming the simulation results. The observed increase in the standard deviation from the mean value in these NWs can be attributed to imperfect contact between some NWs and the gold substrate caused, for example, by roughness of the gold surface or accidental stacking of two NWs on top of each other preventing contact with the gold. We, therefore, expect that the TS-TPA efficiency can be increased further by improving the NW-gold interface. The obtained results underline the importance of optimizing the electric field distribution of the excitation light in designing efficient NW upconverters.

### Comparison with Other Upconverting Materials

To evaluate the UCE of our approach using heterostructured NWs in the context of the state-of-the-art by other approaches, we compare the obtained UCE values with results reported in other upconverting materials (Table 2), including III–V materials, lanthanide-doped nanocrystals, TTA molecules, as well as colloidal II–VI quantum dots (QD). In some references, the *I*_UPL_ and *I*_PL_ are not reported, whereas the internal upconversion quantum yield (UCQY) is instead specified, that is, the ratio of upconverted to absorbed photons. It is clear from Table 2 that the UCE achieved by our approach represents the highest value among semiconductor nanomaterials, by several orders of magnitude as compared to for example, InAs QDs,^33^ defect centers in NWs,^31^ and II–VI colloidal QDs.^34^ This is especially true at low excitation powers, owing to the lower power factors *k* = 1.1 and 1.3 characteristic for the NW heterostructures studied in this work. This can be attributed to a higher density of the involved intermediate states, that is, the band states of the lower-bandgap region. The observed independence of the UCE on the excitation power in our heterostructured NWs means that this upconversion process is well suited for low-power applications.

**Table 2:** A Comparison of the UCE and UCQY between the Present Work and Previously Reported Record Values for Other Material Systems, Measured under the Specified Power Densities and Power Factors^a^

material	UCE (*I*_UPL_/*I*_PL_) (%)	internal UCQY (%)	power density (mW/cm^2^)	power factor, *k*, of UPL	ref
GaAsP/GaNAsP NWs (on Au)	1.3 (6.7)		100	1.1	this work
GaAs/GaNAs NWs	0.2		100	1.3	this work
InAs QDs in GaAs	0.08*		100	1.7	(33)
defects in GaNP NWs	0.07*		100	1.3	(31)
CdSe(Te)/CdSSe/CdSe colloidal QDs	0.02*	0.0008	1000	2.0	(34)
triplet–triplet annihilation molecules		35.2	30	2.0	(61)
lanthanide-doped nanocrystals		3.5	78000	2.0	(18)

a The power factor (*k*) indicates the excitation power (*P*) dependence of the UPL intensity, *I*_UPL_ = *CP*^*k*^, *C* is a constant. The values marked with * are extrapolated using the reported power dependencies to give comparable power densities.

We should note that the upconversion efficiency in the TTA molecules^61^ and lanthanide-doped nanocrystals^18^ has demonstrated higher values than that achieved in this study. However, semiconductors have several advantages that are superior to other material systems and should be taken into account when benchmarking different materials for applications. For example, they provide a wide absorption bandwidth and a band alignment where photons of different energies may be absorbed in the upconversion process as well as a large spectral tunability through alloying, which provides further advantages compared to TTA molecules and lanthanide-doped crystals with generally fixed absorption energies. Semiconductor NWs also allow integration with existing Si-based technologies,^20−22^ which can combine the optoelectronic functionality of the III–V semiconductors with the nanoelectronic functionalities of Si. We should also note that the heterostructured semiconductor NWs provide a large dynamic power range for photon upconversion as, besides low-power operation discussed above, they can also operate under very high excitation powers without suffering photobleaching or other damage thanks to inherent hardness of these materials. These advantages underscore the potential impact of this material system for future applications in nanophotonics or next-generation photovoltaics.

**Table 3:** Structural Parameters of the Investigated NWs^a^

structure	*t*_core_ (nm)	*t*_shell_ (nm)	[N] (%)	[P] (%)	*E*_g_^core^ (eV)	*E*_g_^shell^ (eV)
GaAs	400	–	0	0	1.52	–
GaAs/GaNAs	100	100	0.4	0	1.52	1.40
GaAsP	90	–	0	24	1.81	–
GaNAsP	110	–	0.1	24	1.70	–
GaAsP/GaNAsP*	50	75	1.1	24	1.81	1.50
GaAs/GaNAs/GaAs*	100	30/130	0.3	0	1.52	1.42/1.52

a *t*_core_ (*t*_shell_) denotes the radial thickness of the core (shell) region within the NW heterostructure, [N] and [P] are the fractions of nitrogen [N] and phosphorus [P] atoms in the group-V sublattice, respectively, and *E*_g_^core^ (*E*_g_^shell^) is the band gap energy of the core (shell) material. The structures marked with * were used in the TS-TPA experiments. In the case of the GaAs/GaNAs/GaAs core/multishell NWs, the parameters *t*_shell_ and *E*_g_^shell^ are specified for the N-containing inner/outer shells, respectively.

## Conclusions

In summary, we have demonstrated a type of efficient upconverting semiconductor nanostructure, namely a core/shell NW heterostructure consisting of a nitrogen-free GaAs(P) core and a dilute-nitride GaNAs(P) shell with a smaller bandgap. By monitoring the PL emission of the NW core with a larger bandgap, we have shown that a dramatic enhancement of the upconversion efficiency, which is about 500-fold in the GaAsP/GaNAsP and 100-fold in the GaAs/GaNAs core/shell NWs, is observed when the excitation photon energy is tuned within the range of band-to-band transitions in the dilute-nitride shell. The revealed upconversion process exhibits a nearly linear dependence on the excitation power and, therefore, can be detected at very low excitation densities, *W*_exc_, down to 0.1 W/cm^2^, which is comparable with 1 sun illumination. We have provided compelling experimental evidence that identifies TS-TPA via the band states of the dilute-nitride shell as the dominant mechanism for the observed upconversion, which is promoted in the studied NWs by a favorable band alignment. Based on the performed rate equation analysis supported by the transient PL measurements, the upconversion efficiency is shown to be strongly dependent on the carrier lifetime in the NW shell, reaching 1.35% in the GaAsP/GaNAsP NW arrays fabricated on a Si substrate, a value which is almost independent of the excitation power density. The UCE value can be further enhanced to up to 15% in hybrid NW-on-gold structures, where the electric field distribution is engineered to maximize light absorption within the shell region under upconversion conditions. This is in combination with an overall increase in the emission intensity of these structures caused by a decreased leakage of the laser light outside the NWs. The upconversion efficiency of the core/shell NW heterostructures substantially exceeds those reported for other semiconductor nanostructures, which demonstrates the great potential of dilute-nitride NWs as energy upconverters in, for example, nanophotonic or next-generation photovoltaic applications. Our findings also provide general guidelines for designing efficient nanoscale photon upconverters based on NW heterostructures.

## Experimental Section

### Samples

All investigated NW structures were grown on (111) Si substrates using plasma-assisted molecular beam epitaxy (MBE) with Ga droplets as a self-catalyst. In the case of GaAs and GaNAs NWs, the surface of the Si substrates was not treated prior to the NW growth and, therefore, was covered by a native oxide. NW nucleation occurred at spontaneously formed pinholes in the native oxide leading to a lower yield of vertically aligned NWs. In the case of the GaAsP and GaNAsP NW samples, the native oxide was removed by HF etching prior to NW growth, followed by rinsing the Si substrate with deionized water and its annealing at 710 °C for 15 min. Ga atoms were then deposited on the Si substrate for 1 min with a Ga flux of around 0.7 monolayer/s. The subsequent substrate annealing created Ga droplets, acting as a catalyst for the NW growth. It has been demonstrated that such pretreatment of the substrate gives rise to a higher yield of vertically aligned NWs.^62−64^

For the growth of phosphorus-free NWs, solid elemental Ga and As sources were used, while for the structures containing phosphorus, As and P were provided from thermally cracked AsH_3_ and PH_3_, respectively. Nitrogen was supplied from an rf plasma. In the case of uniform (i.e., not core/shell) NWs grown via the VLS mechanism, the NW diameter was controlled by the size of the seed particles. Radial heterostructure NWs were fabricated by first forming a NW core using the VLS technique and then switching the growth mode to radial vapor–solid (VS) growth to form a shell. In this case, the total NW diameter is controlled primarily by the growth time of the VS-grown shell. For the core/shell NW structure, similar thicknesses could then be obtained for both P-free and P-containing structures. A detailed description of the growth conditions and structural characterization of the Ga(N)As-based and the Ga(N)AsP-based NWs can be found elsewhere.^65,66^ The intended As/P-ratio in the Ga(N)AsP NWs was verified by energy dispersive spectroscopy combined with temperature-dependent PL and Raman measurements.^67^ The N content was estimated using the band anticrossing model,^36^ based on the bandgap energies of the dilute-nitride alloys deduced from temperature-dependent PL and PLE data.^67^ The presence of nitrogen in the NW lattice was further confirmed by the appearance of the Ga–N vibrational mode (LO_2_) in Raman spectra of the dilute-nitride NWs.^67^ Structural parameters of the studied NWs are summarized in Table 3, whereas representative SEM images of the NW arrays are shown in Figures S1 and S2 of the Supporting Information. The SEM images were acquired using a Zeiss Sigma 300 scanning electron microscope operating with an extraction voltage of 2–4 kV. In all cases, the NWs are found to form rather dense arrays. The yield of the vertical NWs within the arrays was higher for the GaAsP-based structures, as expected due to the pretreatment of the Si substrates. This change in the vertical yield, however, does not affect the structural properties of the NWs. According to our previous transmission electron microscopy (TEM) studies^39,55,67,68^ all NWs sampled by TEM have predominantly zincblende crystal structure with the NW axis oriented along the [111] crystallographic direction.

### Methods

For the TPA experiments shown in Figure 1, the NW arrays were mounted in a closed-loop He cryostat cooled to 7 K. A wavelength tunable Ti:sapphire laser operating in the pulsed mode (76 MHz, 150 fs pulse width) was used for PL excitation. For above bandgap excitation, the laser was used in combination with a second-harmonic generation crystal to double the frequency of the light. The PL light was dispersed in a double grating monochromator and detected by a Si avalanche photodiode. In the TS-TPA experiments (Figure 2), a cw 532 nm solid-state laser diode and a Ti:sapphire laser in the cw mode (tuned to a wavelength of 710 nm) were used for one-photon excitation. The Ti:sapphire laser was also used in the UPL and PLE measurements. Time-resolved PL measurements (Figure 3) were conducted at 7 K on NW arrays. As an excitation source, a pulsed frequency doubled Ti:sapphire laser operating at 410 nm was employed. The transient PL signal was detected using a streak camera attached to a single grating monochromator. In all structures, PL and UPL signals solely originate from the NWs, as no emission could be detected from undergrowth particles seen in the SEM images of the NWs arrays.

To measure the emission spectra of single NWs (Figure 5), the NWs were first mechanically transferred to gold and SiO_2_ substrates and then placed in a coldfinger cryostat operating at 5 K. The individual NWs could be resolved in an optical microscope using a 50× 0.5 NA objective, which was also used to focus the excitation light and collect the PL signal from each NW. A cw Ti:sapphire laser and a cw 532 nm solid-state laser were used as excitation sources for below and above shell bandgap excitation, respectively. The PL signal was dispersed using a monochromator and detected by a Si CCD camera.

In all upconversion measurements, an appropriate long-pass optical filter was placed in the path of the excitation beam to prevent any unwanted high-energy light from reaching the sample.

## Supporting Information Available

Electron microscopy images. Effects of the NW geometry on the upconversion efficiency. Analysis of PL transients. Analysis of factors limiting the upconversion efficiency. Effects of enhanced light confinement in NW-on-gold structures. Consideration of strain and piezoelectric fields (PDF)
