# Supplementary material for: Designing Semiconductor Nanowires for Efficient Photon Upconversion via Heterostructure Engineering
Source: ACS Nano. 2022 Jul 25;16(8):12666–76. doi: 10.1021/acsnano.2c04287 (PMC9413407; doi:10.1021/acsnano.2c04287)
Supplement: Supplementary file 1 — nn2c04287_si_001.pdf [file nn2c04287_si_001.pdf]

Supporting information

Designing Semiconductor Nanowires for Efficient Photon Upconversion *via* Heterostructure Engineering

Mattias Jansson[1,], Fumitaro Ishikawa[2,†], Weimin M. Chen[1], Irina A. Buyanova[1,*]*

[1]Department of Physics, Chemistry and Biology, Linköping University, SE-58183 Linköping, Sweden

[2] Graduate School of Science and Engineering, Ehime University, 790-8577, Matsuyama, Japan

[*] Email: mattias.jansson@liu.se, irina.bouianova@liu.se

[†]Present Address

Research Center for Integrated Quantum Electronics, Hokkaido University, Sapporo 060-8628, Japan

S.1. Scanning electron microscopy

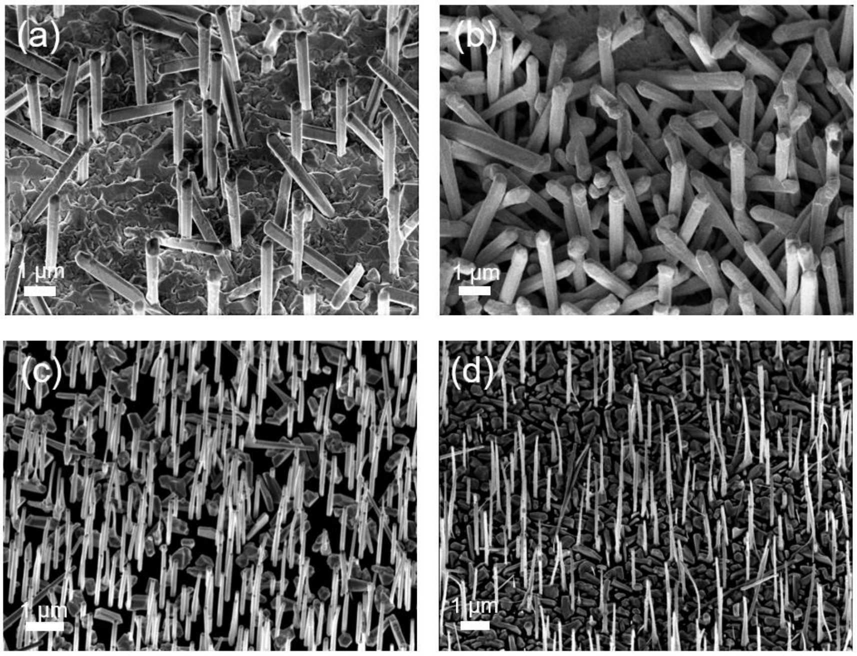

Figure S1. SEM images of the samples used in the two-photon absorption (TPA) studies: (a) GaAs/GaNAs core/shell nanowires (NWs) with [N]=0.4%, (b) GaAs NWs, (c) GaNAsP NWs with [N]=0.1% and [P]=24%, and (d) GaAsP NWs with [P]=24%.

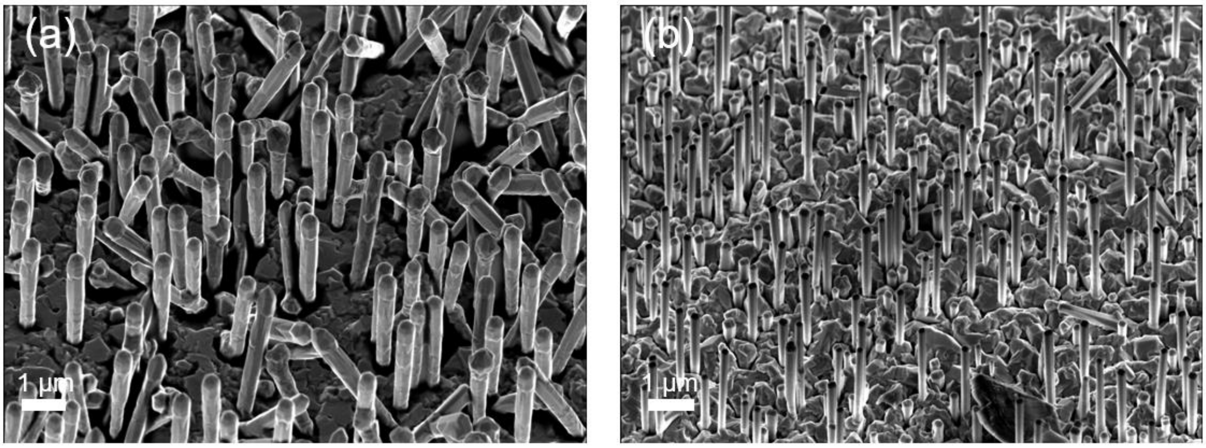

Figure S2. SEM images of the samples used in the two-step (TS)-TPA studies: (a) GaAs/GaNAs/GaAs core/shell/cap NWs with [N]=0.3%, and (b) GaAsP/GaNAsP core/shell NWs with [N]=1.1% and [P]=24%.

S.2. Effects of nanowire geometry on two-photon absorption via virtual states

In this section, we address the possible impact of the NW morphology on the upconversion efficiency (UCE) of the TPA process via virtual states. In principle, several effects could influence this process. First of all, the non-linear response determined by the third-order susceptibility tensor could be affected if vertical and tilted NWs have different crystalline structures and/or different crystallographic orientations of the NW axes. However, according to our previous transmission electron microscopy (TEM) studies,[1-4] this is not the case. All NWs sampled by TEM have predominantly zinc-blende crystal structure with the NW axis oriented along the [111] crystallographic direction. Therefore, we do not expect any difference in the non-linear response caused by the third-order susceptibility tensor between the vertically aligned and tilted NWs that would otherwise affect their TPA response. Secondly, the TPA process may be affected by a difference in light absorption between the vertical and tilted NWs. Indeed, a dielectric mismatch between NWs and the surrounding matrix may suppress absorption of light orthogonally polarized relative to the NW axis. This would degrade the efficiency of non-linear optical processes such as TPA in vertically aligned NWs where the polarization of the excitation light is theoretically perpendicular to the NW axis. Such an effect, however, is known to be important only in thin NWs. Therefore, to examine if this effect is chiefly responsible for the observed differences in the TPA efficiency (Figure 1d of the main text), we performed additional measurements using thicker GaAsP/GaNAsP NWs with d=200 nm. It is found that the upconversion efficiency of the TPA process is comparable in both thin and thick NWs of the same material, see Figure S3. This could be due to effective scattering of the excitation light within the NW array, which randomizes its polarization prior to light absorption from the side of the NWs. We, therefore, conclude that the differences in the NW orientation and thickness do not have a decisive effect on the upconversion efficiency of the studied NWs. Instead, it is the difference in the alloy compositions that plays the dominant role.

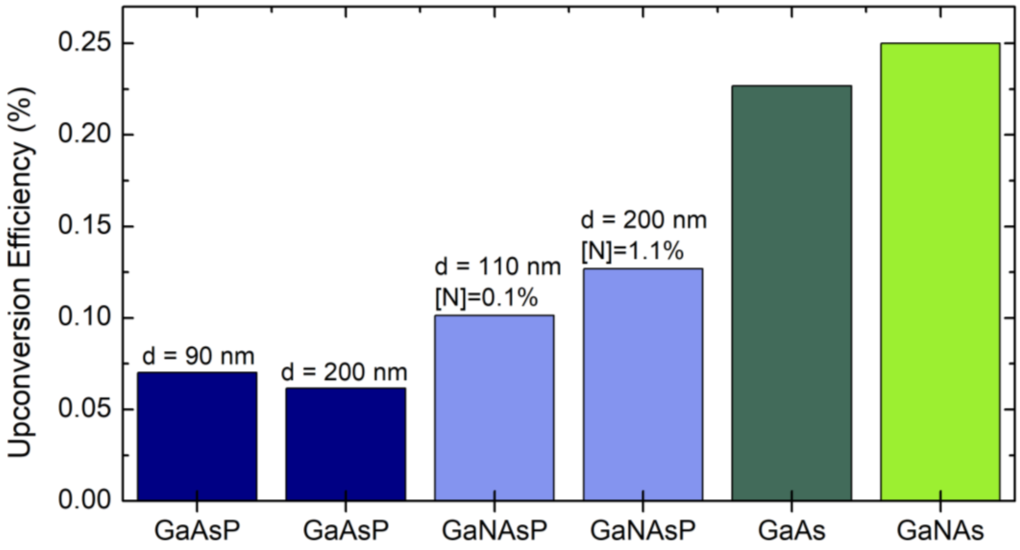

Figure S3. TPA upconversion efficiency through a virtual state, measured at P=0.8 μJ/cm^2/pulse and T=7 K.

S.3. Time resolved photoluminescence spectroscopy

PL decay of the shell emission (shown by the symbols in Figure S4) can be fitted by a bi-exponential function (the solid lines in Figure S4), which contains slow and fast decay components,

$$I = C_f e^{-t/\tau_f} + C_s e^{-t/\tau_s}. \qquad (S1).$$

Here $C_f\,(C_s)$ and $\tau_f\,(\tau_s)$ are the amplitude and the decay time constant of the fast (slow) decay component, respectively. For easy reference, these individual components are shown in Figure S4 by the dotted lines. Such behavior is typical for dilute nitride NWs and likely reflects contributions of radiative transitions from regions with distinctly different lifetimes, determined by a combined effect of radiative and non-radiative recombination. The latter process can dominate in the NW regions with a high density of defects acting as efficient non-radiative recombination centers, e.g. surface states of the NW, interfacial defects at the core/shell NW heterojunction, and point or structural defects.[4-6] The parameters yielding the best fits are given in Table S1. Comparison of the deduced parameters shows that the slow component dominates in the shell emission.

Table S1. The amplitudes and lifetimes of the fast (f) and slow (s) decay components of the shell emission.

Sample	C_f	C_s	τ_f (ns)	τ_s (ns)
GaAs/GaNAs	0.35	0.70	0.4	8.5
GaAsP/GaNAsP	0.4	0.64	1.3	21.0

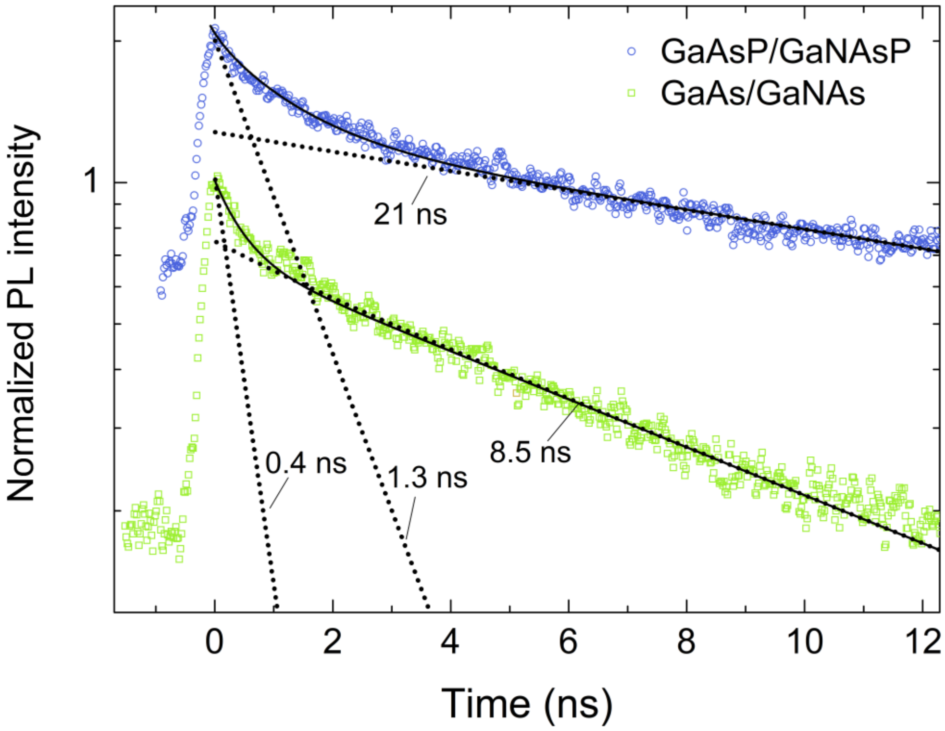

Figure S4. Temporal profiles of the integrated shell emission measured at 7 K from the GaAs/GaNAs (the open green squares) and GaAsP/GaNAsP (the open blue circles) core/shell NWs, under excitation with the wavelength 410 nm. The solid lines show the best fit to the data by a bi-exponential function, with the parameters given in Table S1. The dotted lines show the two individual components of the bi-exponential PL decays.

S.4. Limiting factors of the upconversion efficiency

To understand what the main factors limiting the UCE are, we performed simulations of the UCE as a function of the shell lifetime, τ_{shell}, and upconversion rate, γ, spanning over several orders of magnitude, with the aid of the rate equation model described in the main text. The simulation results are shown in Figure S5, where the stars indicate the simulation results using the parameters experimentally determined for the GaAsP/GaNAsP NWs. We find that both

τ_{shell} and γ dramatically affect the upconversion efficiency and should be optimized to enhance it.

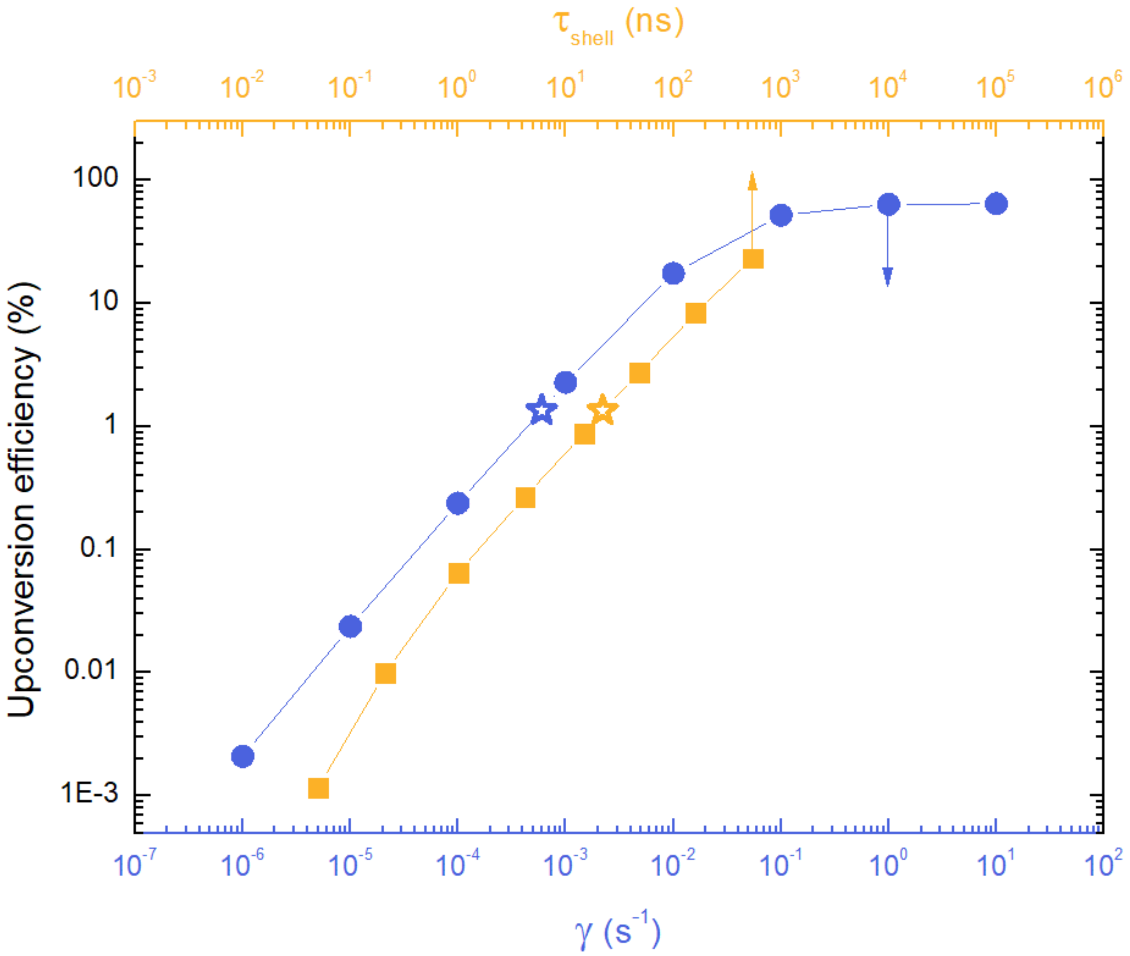

Figure S5. Simulated upconversion efficiency as a function of the shell lifetime, τ_{shell}, (the yellow squares) and upconversion rate, γ, (the blue circles). The constant parameters used in these simulations are the same as those used in the rate-equation simulations described in the main text (see Table 1, first row). The stars indicate the simulations results using the shell lifetime and upconversion rate that correspond to the experimentally measured values in the GaAsP/GaNAsP NWs.

S.5. Effects of enhanced light confinement in NW-on-gold structures

In Figure 5 of the main text, we show how a gold substrate causes a re-distribution of the excitation light inside a NW leading to its better spatial overlap with the shell layer and, therefore, to an increase in the upconversion efficiency. Besides this effect, the FDTD

simulations also show that the gold substrate causes an overall increase in the total light confinement in the NW. By integrating the simulated squared electric field $|E|^2$ over the total NW cross-sections we find that it is 1.9 times higher on gold as compared to SiO_2 when $E_g^{shell} < h\upsilon_{exc} < E_g^{core}$. The corresponding value under the condition of $h\upsilon_{exc} > E_g^{core}$ is 1.2. This increase in confinement of the electric field is expected to give rise to increased light absorption, and therefore also higher PL intensities (I) under one-photon (OP) and TS-TPA excitation. Since in both cases the PL intensity increases approximately linearly with the excitation power, i.e.

$$I_{OP} \propto |E|^2, \quad \text{(S2)}$$

$$I_{TS-TPA} \propto |E|^2, \quad \text{(S3)}$$

we find that the increased light confinement in the NW accounts for an increase of the UCE of

$$\frac{I_{TS-TPA}^{Au}}{I_{OP}^{Au}} = \frac{1.9}{1.2} \approx 1.6. \quad \text{(S4)}$$

Here the superscripts indicate the gold substate. Similarly, the efficiency of TPA via virtual states is also expected to increase. Since the TPA luminescence intensity increases quadratically with excitation power as

$$I_{TPA} \propto (|E|^2)^2, \quad \text{(S5),}$$

the TPA efficiency is expected to increase by a factor of

$$\frac{I_{TPA}^{Au}}{I_{PL}^{Au}} = \frac{1.9^2}{1.2} \approx 3. \quad \text{(S6)}$$

S.6. Consideration of strain and piezoelectric fields

Lattice mismatch between a nitrogen-free core and a dilute nitride shell in a core/shell NW heterostructure will cause a strain in the NW, which, in turn, can lead to the appearance of a piezoelectric field. This field could potentially affect the upconversion process. In a core/shell NW, the longitudinal strain ϵ_{zz} in the core and shell regions is given by[7]

$$\epsilon_{zz}^{core} = (1-\eta)f, \quad \text{(S7)}$$

$$\epsilon_{zz}^{shell} = -\eta f, \quad \text{(S8)}$$

where z is defined along the NW axis, f is determined by the mismatch of lattice constants, a, as

$$f = \frac{(a_{\text{shell}} - a_{\text{core}})}{a_{\text{core}}}, \tag{S9}$$

and η is the ratio of the core and shell cross-sectional areas. Similarly, the in-plane (i.e. orthogonal to z) strain components in the radial (r) and angular (θ) directions are given by

$$\epsilon_{\theta\theta}^{\text{core}} = \epsilon_{rr}^{\text{core}} = (1 - \eta)(f + B_s), \tag{S10}$$

$$\epsilon_{\theta\theta}^{\text{shell}} = B_S \frac{r_{\text{core}}^2}{r^2} - \eta(f + B_s), \tag{S11}$$

$$\epsilon_{rr}^{\text{shell}} = -\eta(f + B_s) - B_S \frac{r_{\text{core}}^2}{r^2}, \tag{S12}$$

where r is the radius, r_{core} is the total radius of the core layer and

$$B_s = -f \frac{c_{11} + 2c_{12}}{c_{11} + c_{12} + 2c_{44}}. \tag{S13}$$

Here c_{11}, c_{12} and c_{44} are the elastic constants. For the GaAsP/GaNAsP structure, we obtain $\epsilon_{zz}^{\text{core}} = -1.8 \cdot 10^{-3}, \epsilon_{zz}^{\text{shell}} = 1.3 \cdot 10^{-4}, \epsilon_{\perp}^{\text{core}} = -4.0 \cdot 10^{-4}, \epsilon_{\perp}^{\text{shell}} = 2.7 \cdot 10^{-5}$, where $\epsilon_{\perp} = (\epsilon_{\theta\theta} + \epsilon_{rr})/2$ represents the total in-plane strain. It is clear that the longitudinal uniaxial strain, ϵ_{zz}, dominates both in the core and shell regions of the NW. Similarly, the strain may be calculated for a core/shell/shell NW, where the longitudinal strain is also shown to dominate.[8]

In a zincblende material, a uniaxial strain along the [111] direction gives rise to a piezoelectric field, E_p:[9]

$$E_p = \frac{2e_{14}\epsilon_{zz}}{\sqrt{3}\varepsilon_0\varepsilon_r}, \tag{S14}$$

directed along the [111] direction, i.e. parallel to the NW axis. Here, e_{14} is the piezoelectric coefficient, and ε_0 and ε_r are the vacuum permittivity and the dielectric constant, respectively. This field is directed parallel to the NW axis, and will therefore not affect the movement of the carriers in the direction of the heterojunction, which means that E_p is not expected to affect the upconversion efficiency. In zincblende core/shell NWs, a small shear strain is expected to appear,[7] which will also give rise to a piezoelectric field in the plane orthogonal to the NW axis, which may cause a carrier drift towards/away from the heterojunction. However, this field is expected to be small compared to the axial field and, therefore, would only marginally affect the upconversion properties.[10] Experimentally, we do not see any contribution of piezoelectric fields on the upconversion properties, since the same power dependence of the UPL intensity was observed within the wide range of the excitation

powers used, while a piezoelectric field is expected to be screened at high excitation powers, causing a change in the power dependence.

References

[1] Filippov, S.; Jansson, M.; Stehr, J. E.; Palisaitis, J.; Persson, P. O. Å.; Ishikawa, F.; Chen, W. M.; Buyanova, I. A. Strongly Polarized Quantum-Dot-Like Light Emitters Embedded in GaAs/GaNAs Core/Shell Nanowires. *Nanoscale* **2016**, *8*, 15939.

[2] Jansson, M.; Chen, S.; La, R.; Stehr, J. E.; Tu, C. W.; Chen, W. M.; Buyanova, I. A. Effects of Nitrogen Incorporation on Structural and Optical Properties of GaNAsP Nanowires. *J. Phys. Chem. C* **2017**, *121*, 7047.

[3] Yukimune, M.; Fujiwara, R.; Ikeda, H.; Yano, K.; Takada, K.; Jansson, M.; Chen, W. M.; Buyanova, I. A.; Ishikawa F. GaAs/GaNAs Core-Multishell Nanowires with Nitrogen Composition Exceeding 2%. *Appl. Lett.* **2018**, *113*, 011901.

[4] Jansson, M.; Francaviglia, L.; La, R.; Balagula, R.; Stehr, J. E.; Tu, C. W.; Fontcuberta i Morral, A.; Chen, W. M.; Buyanova, I. A. Increasing N Content in GaNAsP Nanowires Suppresses the Impact of Polytypism On Luminescence. *Nanotechnology*, **2019**, *30*, 405703.

[5] Chen, S. L.; Chen, W. M.; Ishikawa, F.; Buyanova, I. A. Suppression of Non-Radiative Surface Recombination by N Incorporation in GaAs/GaNAs Core/Shell Nanowires. *Sci. Rep*. **2015**, *5*, 11653.

[6] Amand, T.; Lephay, F.; Valloggia, S.; Voillot, F.; Brousseau, M.; Regreny, A. Exciton Dynamics in GaAs-$Al_xGa_{l-x}As$ Quantum Wells by Picoseconds Time Resolved Photoluminescence Spectroscopy. *Superlattices and Microstructures* **1989**, *6*, 323.

[7] Ferrand, D.; Cibert, J. Strain in Crystalline Core-Shell Nanowires. *Eur. Phys. J. Appl. Phys*. **2014**, *67*, 30403.

[8] Balagula, R. M.; Jansson, M.; Yukimune, M.; Stehr, J. E.; Ishikawa, F.; Chen, W. M.; Buyanova, I. A. Effects of Thermal Annealing on Localization and Strain in Core/Multishell GaAs/GaNAs/GaAs Nanowires. *Sci. Rep.* **2020**, *10*, 8216.

[9] Boxberg, F.; Søndergaard, N.; Xu, H. Q. Photovoltaics with Piezoelectric Core-Shell Nanowires. *Nano Lett.* **2010**, *10*, 1108.

[10] Boxberg, F.; Søndergaard, N.; Xu, H. Q. Elastic and Piezoelectric Properties of Zincblende and Wurtzite Crystalline Nanowire Heterostructures. *Adv. Mater.* **2012**, *24*, 4692.
